# Preventive and therapeutic effects of *Trichinella spiralis* adult extracts on allergic inflammation in an experimental asthma mouse model

**DOI:** 10.1186/s13071-019-3561-1

**Published:** 2019-06-28

**Authors:** Siying Sun, Huihui Li, Yuan Yuan, Liyuan Wang, Wenxin He, Hong Xie, Shifang Gao, Ruoxue Cheng, Haichun Qian, Hui Jiang, Xiaoli Wang, Bin Zhan, Qiang Fang, Xiaodi Yang

**Affiliations:** 1 Department of Microbiology and Parasitology of Bengbu Medical College, Bengbu, 233000 China; 2 Pharmacy College of Anhui Medical University, Hefei, 230001 China; 3 Section of Tropical Medicine, Department of Pediatrics, Baylor College of Medicine, Houston, TX USA; 4 Anhui Key Laboratory of Infection and Immunity, Bengbu Medical College, Bengbu, 233030 China

**Keywords:** *Trichinella spiralis*, Immunomodulation, Asthma, Adult worm and larva extracts

## Abstract

**Background:**

Helminths immunomodulate the host immune system by secreting proteins to create an inhibitory environment as a strategy for survival in the host. As a bystander effect, this balances the host immune system to reduce hypersensitivity to allergens or autoantigens. Based on this, helminth therapy has been used to treat some allergic or autoimmune diseases. As a tissue-dwelling helminth, *Trichinella spiralis* infection has been identified to have strong immunomodulatory effects; the effective components in the worm have not yet been identified.

**Methods:**

The soluble extracts of *T. spiralis* adult worms and muscle larvae were used to treat airway inflammation before and after an ovalbumin (OVA)-sensitization/challenge in an OVA-induced asthma mouse model. The therapeutic effects were observed by measuring the level of inflammation in the lungs.

**Results:**

The soluble products derived from *T. spiralis* parasites, especially from adult worms, were able to ameliorate OVA-induced airway inflammatory responses which were associated with reduced eosinophil infiltration, OVA-specific IgE, Th2 cytokine IL-4, and increased IL-10 and TGF-β. The stimulation of the Treg response may contribute to the alleviated allergic inflammation.

**Conclusions:**

*Trichinella spiralis* worm extracts stimulate regulatory cytokines that are associated with reduced allergic airway inflammation. The identification of effective components in the adult worm extracts will be a crucial approach for developing a novel therapeutic for allergic and autoimmune diseases.

## Background

Allergic and autoimmune disorders represent hypersensitivity and immunopathological reactions to exogenous allergens or to endogenous antigens (autoantigens) [1]. The related diseases, such as asthma and inflammatory bowel diseases, are a significant burden for human health due to their severity, unclear causative factors, and a lack of a cure, drug or vaccine [2].

Since the hygiene hypothesis proposed by Strachan in 1989 [3], an increasing number of experiments and epidemiological studies have revealed the inverse correlation between the autoimmune or allergic diseases with helminth infections [4–8]. This hypothesis suggests that helminth infection shapes the human immune system to reduce its hypersensitivity to allergens or autoantigens [9, 10]. Many experimental studies have provided support for this hypothesis and demonstrated that the immunomodulatory effects of certain helminths and their secreted proteins alleviated not only parasite-specific inflammatory responses that facilitates parasitism, but also other allergic or autoimmune pathology in animal models such as inflammatory bowel disease (IBD), autoimmune encephalomyelitis, arthritis and allergic asthma [11–16]. Since then, clinical trials of helminthic therapy on autoimmune diseases, including inflammatory bowel disease, rhinitis and multiple sclerosis, have been carried out and some extent of therapeutic efficacy has been observed [17–19].

Allergic asthma, a chronic inflammatory disorder of the respiratory tract, has a close relationship with the aberrant Th2 cell responses. It is the Th2 cytokines that result in the symptoms of airway hyperreactivity and increase in mucus production [20]. Although corticosteroids are considered an effective remedy, severe long-term side effects still hinder patients from using them [21, 22]. Helminth therapy with different helminthic species, including *Nippostrongylus brasiliensis*, *Litomosoides sigmodontis* and *Heligmosomoides polygyrus*, has shown to suppress the development of experimental allergic asthma [23–25]. *Schistosoma japonicum* infection also led to a significant decrease in inflammatory cell infiltration around the airway and eosinophil accumulation in bronchoalveolar lavage fluid upon challenge with ovalbumin (OVA) [26]. Some studies demonstrated that the infection of *Trichinella spiralis*, a tissue-dwelling parasitic nematode, could alleviate the manifestation of allergic asthma [27, 28]. However, helminth infections could also cause pathology and disease in humans which raises ethical questions over using a living pathogen for the therapy of other diseases. Usage of certain helminth extracts or derivative instead of using the living parasite for the therapy of allergic and autoimmune diseases has generated substantial interest. The relevant experiments have demonstrated that helminth-secreted molecules [29–32] or their synthetic analogues [33, 34] could reduce the inflammatory responses caused by allergic or autoimmune diseases on the basis of immunoregulatory mechanisms of parasitic worms. Our previous study has shown that the extracts or secreted proteins of *T. spiralis* had therapeutic effects on inflammatory colitis through inducing regulatory T cells [35]. In the present study, we explore the therapeutic effect of the soluble products derived from *T. spiralis* worms on regulating the OVA-specific Th2 responses and reducing the allergen-caused inflammatory reactions in a mouse model, with a final goal to identify those effective worm components as potential for the therapy of allergic and autoimmune diseases.

## Methods

### Animals

Female Balb/c mice 6–8 weeks old were purchased from the Animal Center of Anhui Medical University and maintained in a controlled environment (12:12 h light/dark photocycle with a temperature of 22 ± 2 °C and a relative humidity of 55%).

### Extracts of *T. spiralis* adult worms and muscle larvae

*Trichinella spiralis* ISS 534 strain [36] was maintained in female ICR mice. *Trichinella spiralis* muscle larvae were collected from the muscles of infected ICR mice using a standard pepsin/hydrochloric acid digestion method [37]. The adult worms were collected from the small intestines of Wistar rats experimentally infected with *T. spiralis* muscle larvae for 4 days. After being washed several times in PBS, the soluble adult extracts of (*Ts*-AE) or muscle larvae extracts (*Ts*-MLE) of *T. spiralis* were prepared by conventional methods [38], and the protein concentration was determined by a Bicinchoninic Acid Protein Assay Kit (Beyotime Biotechnology, Shanghai, China).

### OVA sensitization and treatment

**Fig. 1:**
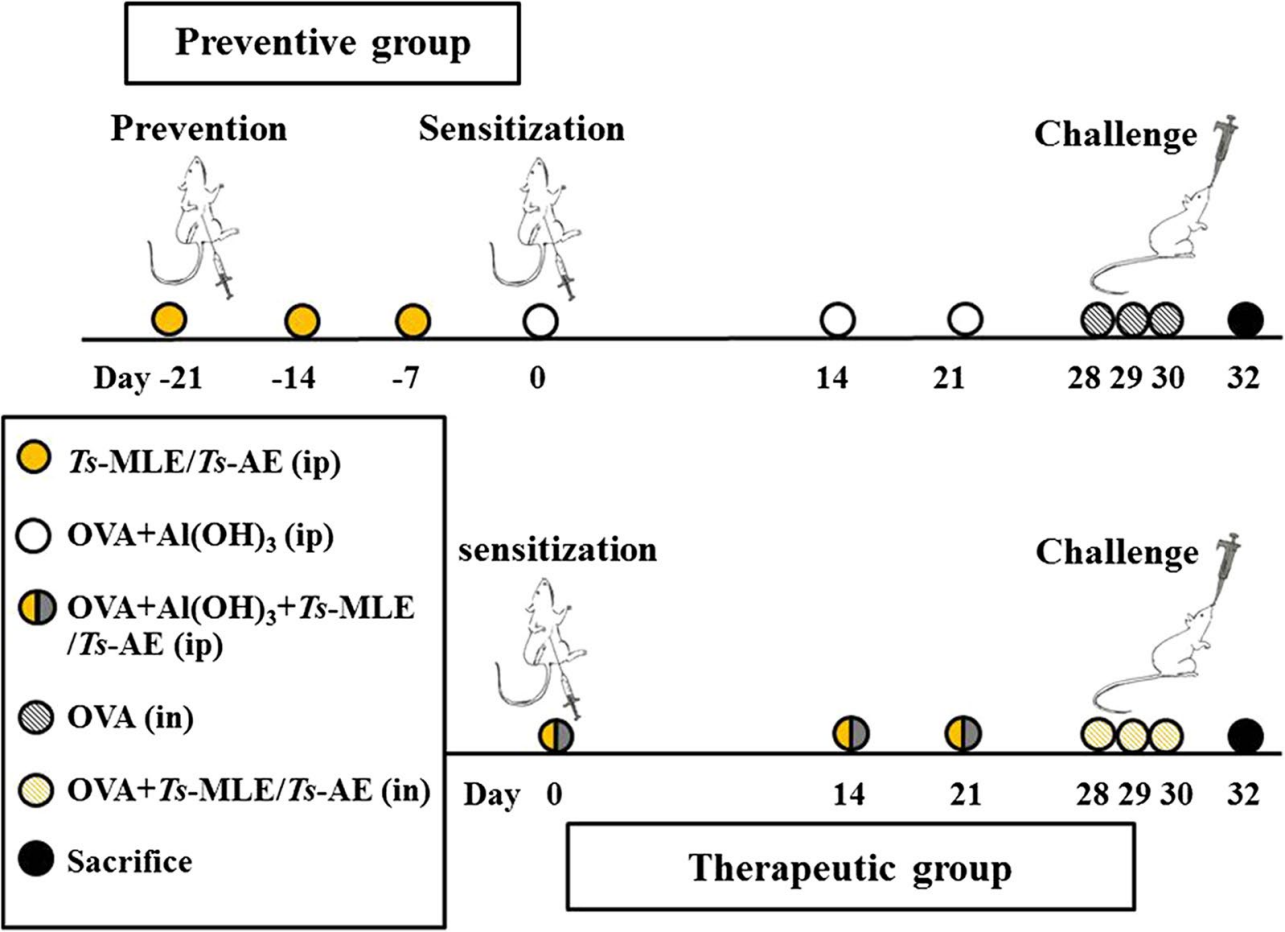
Regimen for mouse OVA sensitization and treatment

Six female Balb/c mice in each group were sensitized with 100 µg of OVA (Sigma-Aldrich, Steinheim, Germany) formulated with 20 μg of Al(OH)_3_ (Sigma-Aldrich) in a total volume of 200 μl by intraperitoneal injection (ip) on day 0, 14 and 21. For preventive groups, mice were treated intraperitoneally with 50 μg of *Ts*-AE or *Ts*-MLE prior to the OVA sensitization on days − 21, − 14 and − 7. For therapeutic groups, mice were co-administrated intraperitoneally with 50 μg of *Ts*-AE or *Ts*-MLE during the OVA sensitization on day 0, 14 and 21. One week after the last sensitization, all mice were challenged intranasally (in) with 100 μg of OVA in a total volume of 50 μl of sterile PBS under sedation of chloral hydrate (8 µg/20 g) for consecutive three days (day 28, 29 and 30) while mice in therapeutic groups were co-administrated intranasally with 25 μg of *Ts*-AE or *Ts*-MLE during the OVA challenge (Fig. 1). One group of mice was OVA-sensitized and challenged without treatment as non-treatment control. Another group of mice without treatment and challenge was used as normal control. Forty-eight hours after the last challenge, all mice were euthanized and the lung tissues were collected for inflammatory assessment, and bronchoalveolar lavage fluid (BALF) and sera were collected for immunological tests. All experiments were repeated once.

### Eosinophil count in bronchoalveolar lavage fluid (BALF)

Forty-eight hours after the last challenge with OVA, all mice were sacrificed and the lungs were lavaged with 0.7 ml of sterile PBS four times. The BALF collected from each mouse was centrifuged at 1500×*g* for 10 min at 4 °C, and the supernatants were stored at 80 °C until use. Cells in precipitate were suspended in 1 ml of PBS stained with Giemsa and Wright’s staining (Solarbio Life Science, Beijing, China) on slides. The eosinophil, lymphocytes, neutrophils and macrophages were counted under a light microscope (Olympus IX71, Olympus, Tokyo, Japan).

### Measurement of cytokines in BALF

The concentration of IL-4, IL-10, TGF-β and IFN-γ in BALF was measured using LEGEND MAX™ ELISA kits (Dakewe Biotech, Beijing, China) according to the manufacturer’s instructions. The optical density (OD) values were read at 450 nm using an ELISA plate reader.

### Analysis of pulmonary histopathology

The lung tissues were fixed with formalin and embedded in paraffin. Sections were cut and stained with hematoxylin/eosin (H&E) (Beyotime Biotechnology). The inflammation of lung tissues was microscopically determined by the degree of cell infiltration around the basal membrane of bronchi or vessels, which were graded on a scale from 1 to 4. A value of 1 was assigned for occasional infiltration of inflammatory cells in lung tissue, a value of 2 was assigned for a thin layer (two to three cells thick) of inflammatory cells, a value of 3 was assigned when bronchi or vessels were surrounded by a thick layer of four to five inflammatory cells, and a value of 4 was assigned when bronchi or vessels were surrounded by a layer of more than five inflammatory cells [39].

### Detection of total IgE and OVA-specific IgE in sera

The levels of total IgE in sera of mice were measured using LEGEND MAX™ ELISA kits (Dakewe Biotech) and OVA-specific IgE measured using Mouse IgE ELISA MAX™ Standard (Biolegend, San Diego, CA, USA) in accordance with the manufacturers’ recommendations.

### Statistical analysis

All data were analyzed for statistical significance (*P* < 0.05) by Student’s two-tailed t-test. One-way analysis of variance (ANOVA) together with the Tukey test for multiple comparisons was used to establish differences between three or more groups.

## Results

### Reduced allergic inflammation in lungs of mice treated with *Ts*-AE and *Ts*-MLE

**Fig. 2:**
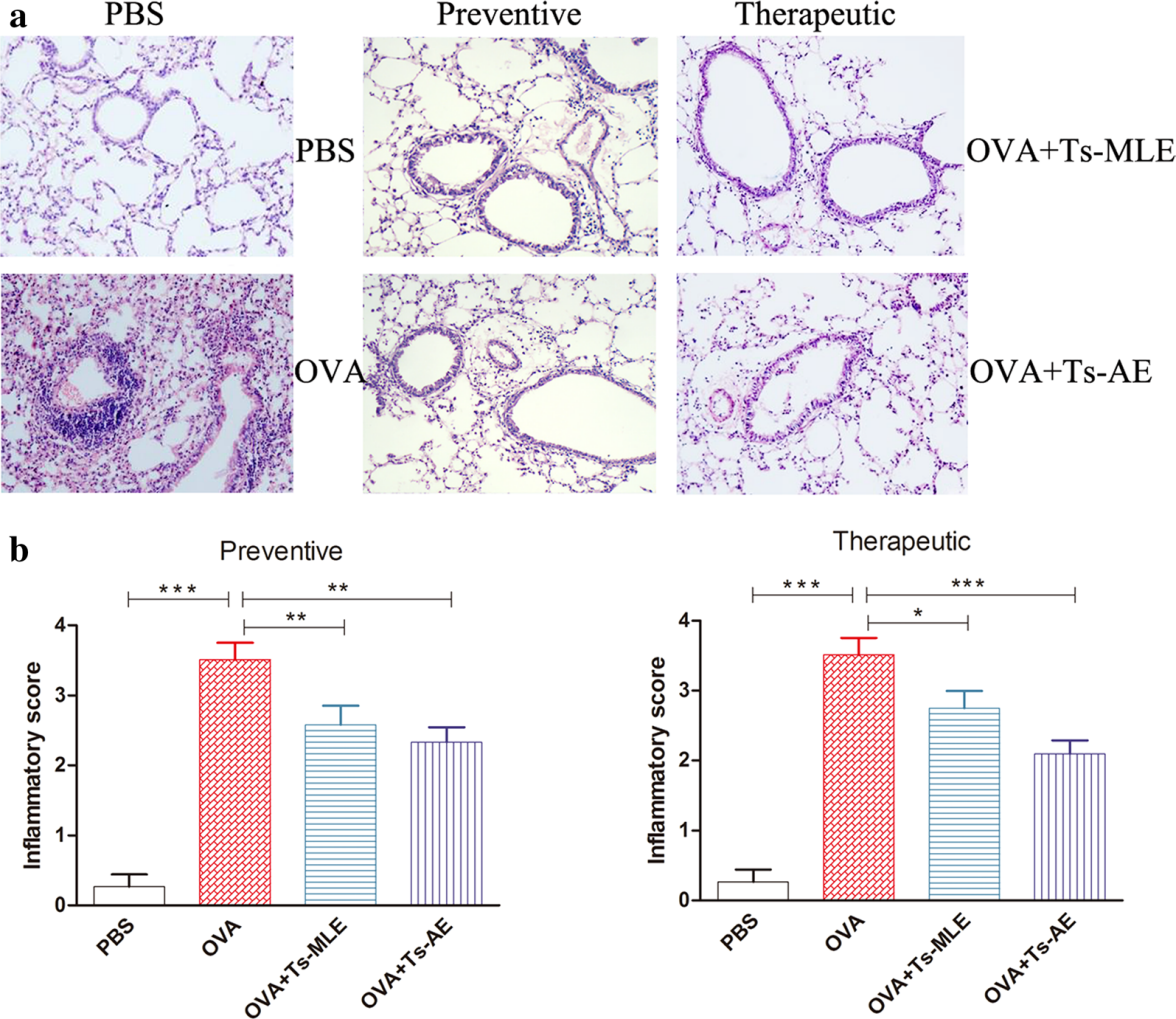
Histopathological changes of pulmonary tissues of OVA-sensitized/challenged mice with treatment of *Ts*-AE or *Ts*-MLE in preventive or therapeutic regimens (×200) (**a**), and the improved inflammatory score of lung tissues with treatment of *Ts-*AE or *Ts*-MLE in preventive or therapeutic regimens (**b**) (*n* = 6 mice per group). *P < 0.05, **P < 0.01, ***P < 0.001

The histochemical observation in lungs of mice sensitized and challenged with OVA showed asthma-like pathology such as significant increased inflammatory cells infiltration, pulmonary edema, and destruction of the alveolar wall compared to the normal control treated with PBS only (Fig. 2). The inflammatory score of lung tissues was significantly increased in OVA-sensitized/challenged compared to the normal control treated with PBS only (ANOVA: *F*_(3,23)_ = 39.34, *P* < 0.0001). However, the lungs of those mice treated with *Ts*-AE or *Ts*-MLE, either in preventive or therapeutic approach, demonstrated significant reduced inflammatory recruitment and immunopathological changes around the airway and the less damaged capillary and vascular endothelial cells. The inflammatory score of lung tissues in groups treated with worm extracts was significantly reduced in contrast to the group without parasite extract treatment (Fig. 2) (ANOVA: *F*_(3,23)_ = 39.34, *P* < 0.0001; *F*_(3,23)_ = 40.59, *P* < 0.0001, respectively). Favorable pathological improvement was more significant in the preventive model than the therapeutic model, with less notable cell infiltration in the former.

### Treatment with *Ts*-AE or *Ts*-MLE reduced eosinophil cells in bronchoalveolar lavage fluid

**Fig. 3:**
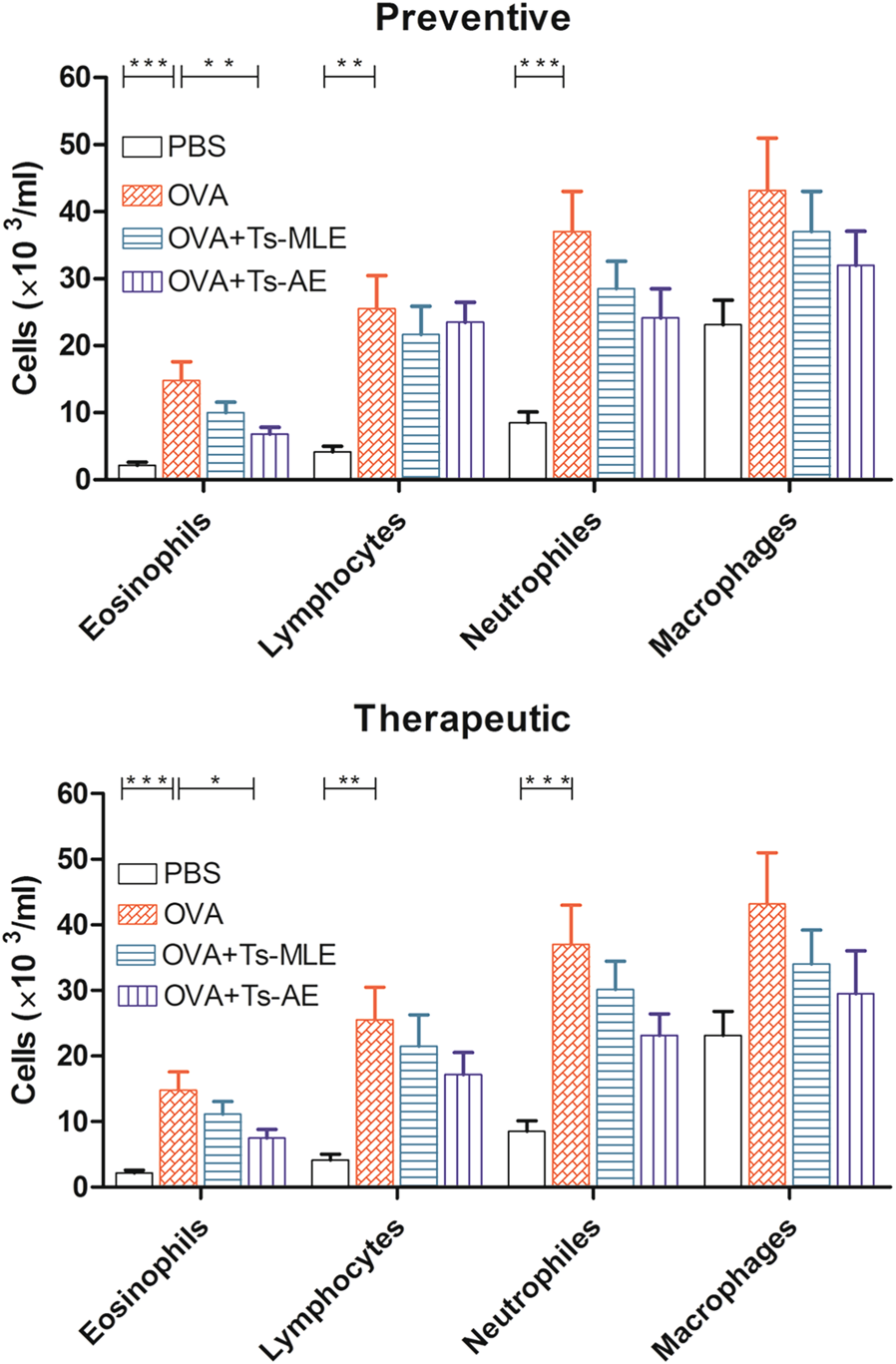
Reduced eosinophil cell count in BALF from mice treated with *Ts*-AE or *Ts*-MLE. Data are expressed as mean ± SEM from two independent experiments (*n* = 6 mice per group). *P < 0.05, **P < 0.01, ***P < 0.001

The eosinophils, lymphocytes and neutrophils were significantly increased in BALF in OVA-sensitized and challenged mice compared to mice without sensitization (with PBS only) (ANOVA: *F*_(3,23)_ = 9.90, *P* = 0.0003; *F*_(3,23)_ = 7.47, *P* = 0.0015; *F*_(3,23)_ = 7.68, *P* = 0.0013, respectively). The reduced eosinophil infiltration in BALF was observed in the groups of OVA-sensitized mice treated with *Ts*-AE and *Ts*-MLE in both preventive and therapeutic regimens. However, the reduced eosinophil cell count in BALF was statistically significant only in groups treated with *Ts*-AE compared to the groups without treatment, not in groups treated with *Ts*-MLE (Fig. 3).

### Treatment with *Ts*-AE or *Ts*-MLE reduced OVA-specific IgE level in OVA sensitized mice

**Fig. 4:**
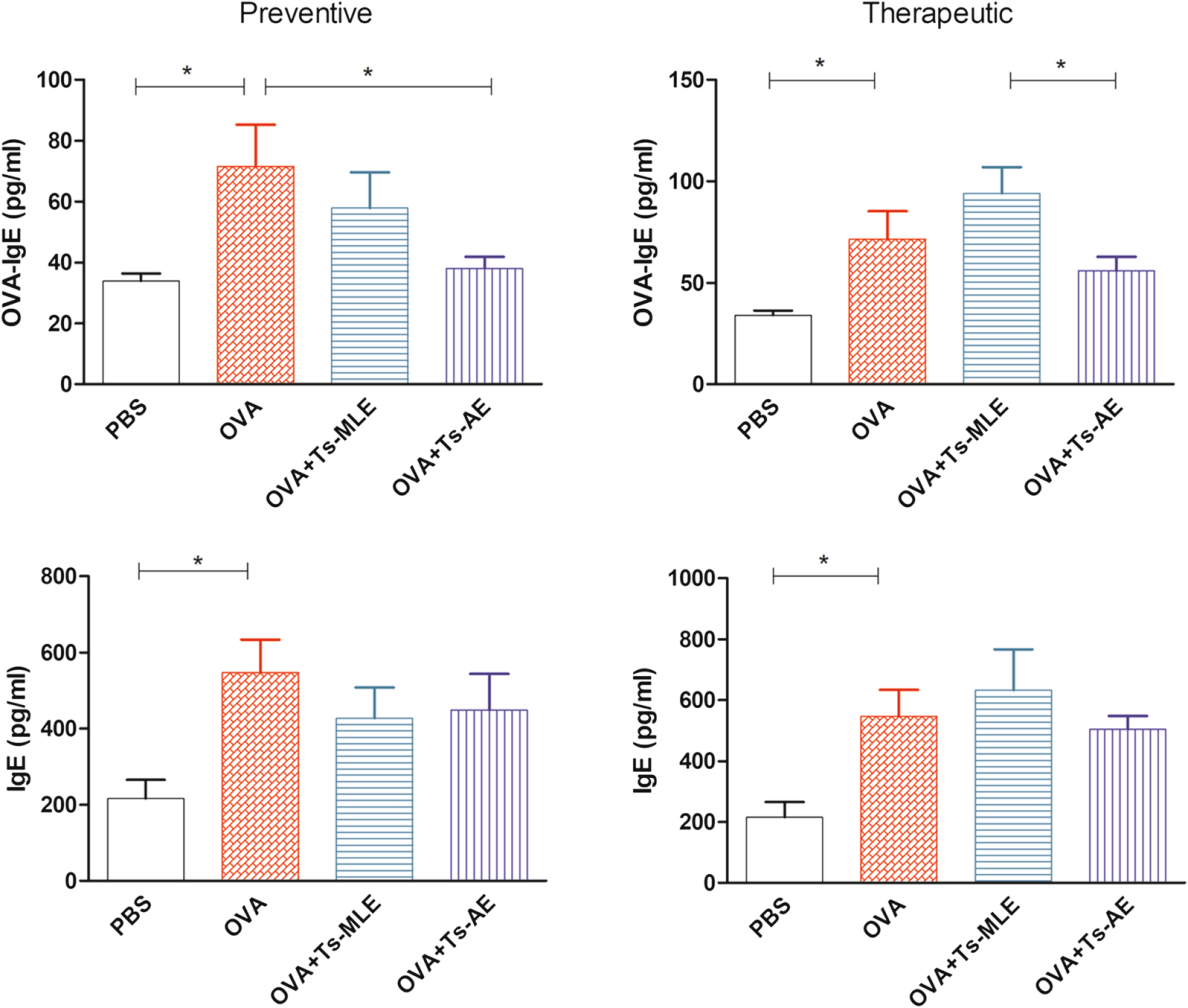
Treatment with *Ts*-AE and *Ts*-MLE reduced the OVA-specific IgE level in sera of mice upon OVA sensitization/challenge. The levels of OVA-specific IgE and total IgE were measured by ELISA in sera of OVA-sensitized mice treated with *Ts*-AE or *Ts*-MLE in preventive and therapeutic models. Data are expressed as mean ± SEM from two independent experiments (*n* = 6 mice per group). *P < 0.05, **P < 0.01, ***P < 0.001

OVA sensitization significantly elevated the levels of both OVA-specific IgE and total IgE in sera of sensitized mice (ANOVA: *F*_(3,23)_ = 3.53, *P* = 0.0335; *F*_(3,23)_ = 2.99, *P* = 0.0554). Treatment with *Ts*-AE or *Ts*-MLE did not change the total IgE level in sera of mice in both preventive and therapeutic models; however, treatment with *Ts*-AE before the OVA sensitization (preventive model) significantly reduced the OVA-specific IgE level in sera of OVA-sensitized mice (ANOVA: *F*_(3,23)_ = 3.53, *P* = 0.0335), but not in the therapeutic model (Fig. 4). The OVA-specific IgE was also reduced in mice preventively treated with *Ts*-MLE before OVA-sensitization, but the reduced level was not statistically significant. This indicates that pre-treatment with *Ts*-AE could reduce the IgE level of the mouse upon OVA sensitization/challenge and that *Ts*-MLE has less ability to reduce the OVA-induced IgE response. After being sensitized with OVA (therapeutic model), *T. spiralis*-derived proteins, both adult and muscle larva, may not be able to reduce the mouse IgE response to OVA sensitization.

### *Trichinella spiralis*-derived proteins inhibit IL-4 and stimulate IL-10 and TGF-β in OVA-sensitized mice

**Fig. 5:**
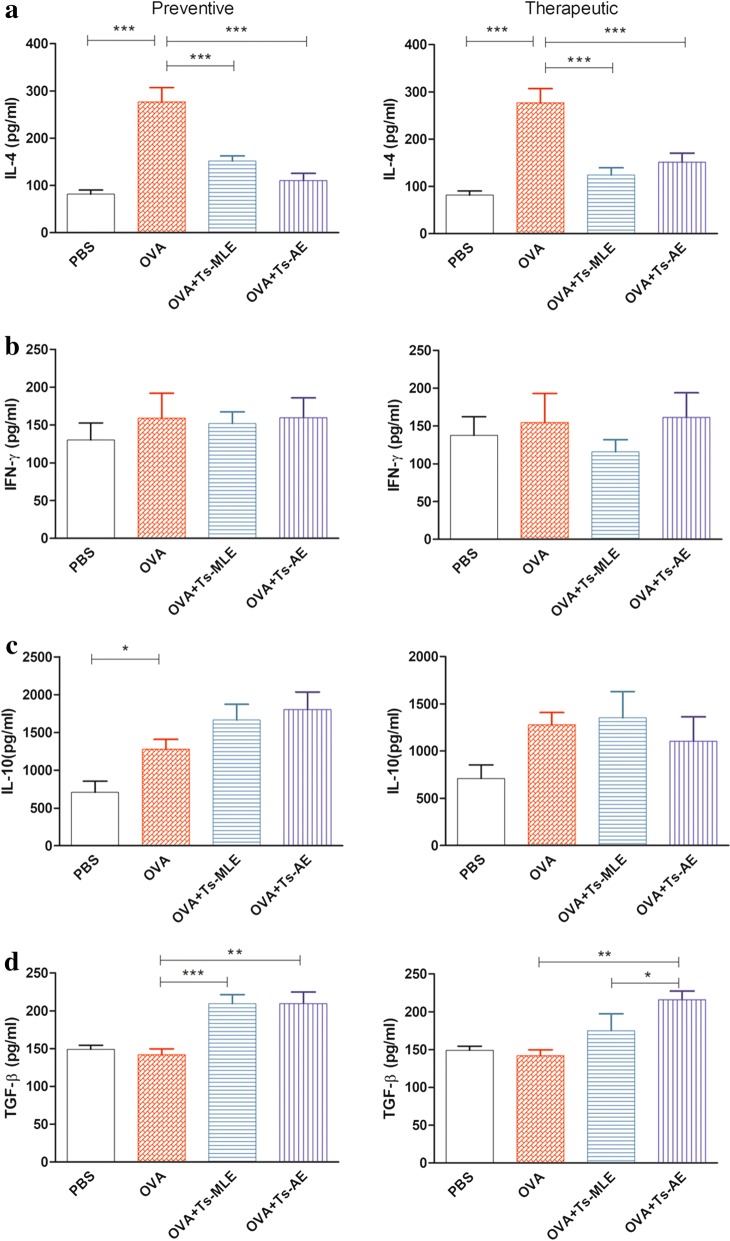
The cytokine levels of IL-4 (**a**), IFN-γ (**b**), IL-10 (**c**) and TGF-β (**d**) were measured in BALF of OVA-sensitized mice upon the treatment of *Ts*-AE and *Ts*-MLE in preventive and therapeutic models. Data are expressed as mean ± SEM from two independent experiments (*n* = 6 mice per group). *P < 0.05, **P < 0.01, ***P < 0.001

Typical cytokines for Th1 (IFN-γ), Th2 (IL-4) and regulatory functions (IL-10 and TGF-β) were measured in the BALF of *T. spiralis*-derived protein treated mice. The secretion of IL-4 in BALF of mice treated with *Ts*-AE or *Ts*-MLE was significantly reduced upon the sensitization/challenge of OVA compared to the groups without treatment in both preventive and therapeutic models (ANOVA: *F*_(3,23)_ = 22.06, *P* < 0.0001; *F*_(3,23)_ = 17.53, *P* < 0.0001) (Fig. 5a). However, the Th1 cytokine IFN-γ did not change upon treatment with any of the parasite proteins in mice sensitized/challenged with OVA (ANOVA: *F*_(3,23)_ = 0.31, *P* = 0.8210; *F*_(3,23)_ = 0.48, *P* = 0.6976) (Fig. 5b). Interestingly, the regulatory TGF-β were significantly boosted in the mice treated with both *Ts*-AE and *Ts*-MLE before OVA sensitization (preventive) (ANOVA: *F*_(3,23)_ = 11.64, *P* = 0.0001). The IL-10 level was increased in OVA-sensitized mice, but boosted in mice preventively treated with both *Ts*-AE and *Ts*-MLE; however, the increased level was not statistically significant compared to groups without treatment. For mice treated with parasite proteins after being sensitized with OVA (therapeutic), only the TGF-β level was increased in *Ts*-AE-treated mice (ANOVA: *F*_(3,23)_ = 6.15, *P* = 0.0039); IL-10 was not boosted for both *Ts*-AE and *Ts*-MLE treatments (ANOVA: *F*_(3,23)_ = 1.813, *P* = 0.1773) (Fig. 5c, d).

## Discussion

To determine whether the worm extracts can be used to alleviate the asthma-caused inflammatory reactions as an alternative for worm infection therapy, the extracts from *T. spiralis* adult worms (*Ts*-AE) or muscle larvae (*Ts*-MLE) were used to treat the OVA-induced asthma before OVA sensitization (preventive) or during sensitization (therapeutic) in a mouse model. The results demonstrated that mice treated with *Ts*-AE before OVA sensitization (preventive) significantly reduced the infiltration of inflammatory cells around the airway and blood vessels in OVA-sensitized mice upon challenge with OVA. The boosted Th2 cytokine (IL-4) and IgE level was seen in this study as typical characteristic of allergic asthma [40]. The immunopathological improvement in the lung tissue in the treated groups is correlated with the reduced eosinophil cells infiltrated in BALF and reduced OVA-specific IgE level in the sera of treated mice compared with groups without treatment. The reduced IgE level is only related to OVA-specific IgE, not the total IgE in the sera. Treatment of *Ts*-AE significantly reduced IL-4 level, increased IL-10 and TGF-β levels in BALF of OVA-sensitized/challenged mice, but had no effect on the level of INF-γ.

In this study, we have demonstrated that the preventive effect of the soluble proteins derived from adult *T. spiralis* on OVA-induced asthma inflammation is associated to the reduced allergen-specific Th2 responses, including reduced OVA-specific IgE in sera and reduced IL-4 level and eosinophil cells in lungs. IL-4 is a typical Th2-inducing cytokine that accelerates the production of IgE stimulated by allergen(s) [41]. The worm-induced high level of worm-specific IgE that may compete with allergen-specific IgE binding sites on mast cells or basophils as part of mechanism in which the helminth infection or helminth-derived proteins are involved in the treatment of allergic and inflammatory diseases [20, 42, 43]. Although asthma is associated with aberrant Th2 responses, the induction of Th1 responses seems to suppress the development of allergic airway inflammation. Treatment with somatic extract of *Caenorhabditis elegans* reduced asthma response with a shift from Th2 to Th1 response in mice [16]. Actually, administration of IFN-γ into the airway before OVA sensitization [44] or after sensitization [45] suppressed the development of OVA-induced airway inflammation. However, we did not find a significant change in the IFN-γ level between groups given with worm extracts and PBS in this study, indicating Th1 may not be involved in the alleviating effect of *Ts*-AE in the asthma model. A similar result also found in another experiment showed that Th1 response was not related to the downregulatory effects of helminthic products on OVA-induced allergic inflammation [20].

As with most helminth infections, treatment with *Ts*-AE increased the levels of IL-10 and TGF-β, indicating the immunomodulation of *Ts*-AE takes place through stimulation of the regulatory pathway of immune system. IL-10 and TGF-β are considered the typical cytokines that confer regulatory function [46, 47] and form an environment of anti-inflammation [48]. CD4^+^CD25^+^Foxp3^+^ regulatory T cells, as well as other immune cells, secret IL-10 and TGF-β [49]. The increased levels of IL-10 and TGF-β upon the treatment with *Ts*-AE in OVA-sensitized mice possibly result from the activation of Treg by the treatment of *Ts*-AE, even though we did not check the presence of Treg in the lung tissue. It is well known that Treg plays important roles in the immunomodulation of host immune system in helminth infections [50]. Treg, induced by *T. spiralis* or other helminth infection or helminth-derived protein, has been identified to be associated to the alleviation of asthma and other inflammatory diseases [51–53]. The Treg-induced suppression of the inflammatory reaction could be adoptively transferred to naïve mice [53]. Depletion of Treg in mice infected with *Schistosoma mansoni* aggravated allergic airway inflammation in an OVA-induced asthma model [39]. Treg is also related to the tolerogenic properties of human monocyte-derived dendritic cells promoted by native *T. spiralis* secreted products [54], which may reduce the hypersensitivity to allergens or autoantigens as one of the mechanisms for helminth-derived proteins to treat or prevent allergic or autoimmune inflammations.

In this study, we also showed that treatment with *T. spiralis* adult worm-derived proteins, instead of worm infection, was able to suppress OVA-induced asthma inflammation. These results suggest that the reduced airway inflammation caused by the infection of *T. spiralis* [27] could be replaced by the application of adult worm extracts; possibly certain active proteins in the adult extracts confer the alleviation of OVA-induced airway inflammation. These results are consistent with our previous study whereby the excretory/secretory products from *T. spiralis* adult worms had a therapeutic potential for alleviating inflammatory colitis in mice. This therapeutic effect was correlated with the upregulation of Treg response and downregulation of pro-inflammatory cytokines [35]. Results in the present study further confirmed that some proteins secreted by adult worms of *T. spiralis* possess anti-inflammatory activities as part of immunomodulatory functions.

Compared to the potent effects of proteins from *T. spiralis* adult extracts on anti-inflammatory activities, the proteins from *T. spiralis* larvae extracts had a less preventive effect on the OVA-induced airway inflammation characterized by the lower effect on the reduction of eosinophil infiltration in BALF and OVA-specific IgE in the sera. The lower therapeutic effect of *T. spiralis* larval extracts on OVA-induced airway inflammation in comparison to adult extracts in this study is consistent with the results of treatment of DSS-induced colitis with *T. spiralis* ES products that showed only adult ES had an effect [35], even though the *T. spiralis* muscle larvae ES products had also shown its inhibitory effect on the dendritic cell maturation and stimulation on the Treg *in vitro* [55].

Even though the *T. spiralis* adult extracts have shown to have preventive effects on the OVA-induced airway inflammation and asthma by the application before OVA sensitization and challenge, many people are usually sensitized by aeroallergens and develop asthma before being exposed to helminth infections or treatment with helminth proteins. Therefore, it is more important to determine whether the helminth-derived products have therapeutic effects on asthma. In this study, we developed a therapeutic model by the treatment of *T. spiralis*-derived proteins (*Ts*-AE or *Ts*-MLE) during OVA sensitization and OVA challenge. We found that both the preventive and therapeutic models had significantly reduced inflammatory infiltration and immunopathological changes, especially with the treatment of *Ts*-AE, although the therapeutic model had a less protective effect with more inflammatory cell infiltration noticed. The lower level of lung tissue improvement in therapeutic model than preventive model is associated with a less reduced OVA-specific IgE level and less increased IL-10 level. A similar effect was observed in a mouse infection model that showed that mice infected with *T. spiralis* before OVA-sensitization and challenge had a better protective effect on airway inflammation than mice infected with *T. spiralis* after being sensitized with OVA [27]. Possibly, treatment with *T. spiralis*-derived proteins stimulates the inhibitory environment that inhibits the allergen sensitization and asthma onset. It has been previously observed that mice infected with *T. spiralis* before OVA-sensitization had a higher number of Treg cells than mice infected after OVA sensitization [27]. After being sensitized by an allergen, it may need more helminth protein treatment or take a longer treatment time to induce enough Treg proliferation and an inhibitory environment for alleviating asthma inflammation.

Our results distinctly show that the soluble products derived from *T. spiralis* parasites, especially from adult worms, can ameliorate airway inflammatory responses in an OVA-induced asthma mouse model. The identification of the effective components in the adult worm extracts will be crucial steps toward improving the therapeutic efficacy, increasing the safety, and facilitating the massive manufacture of helminth protein(s) as novel therapeutic reagents for allergic and autoimmune inflammatory diseases.

## Conclusions

*Trichinella spiralis* worm extracts stimulate regulatory cytokines that are associated with reduced allergic airway inflammation. The identification of the effective components in the adult worm extracts will be a crucial approach for developing novel therapeutics for allergic and autoimmune diseases.

## Data Availability

The datasets generated and/or analyzed during the present study are available from the corresponding author upon reasonable request.

## Abbreviations

*Ts*-AE: soluble extracts of *T. spiralis* adult worms
*Ts*-MLE: soluble extracts of *T. spiralis* muscle larvae
ELISA: enzyme-linked immunosorbent assay
BALF: bronchoalveolar lavage fluid
PBS: phosphate-buffered saline
ES: excretory-secretory
OVA: ovalbumin
Tregs: regulatory T cells
IBD: inflammatory bowel disease
DSS: dextran sodium sulfate
IL-4: interleukin 4
IL-10: interleukin 10
TGF-β: transforming growth factor-β
IFN-γ: interferon γ
